# PVA- Bentonite-Water Coatings: Experimental and Simulation Studies

**DOI:** 10.3390/polym17192689

**Published:** 2025-10-04

**Authors:** Sarojini Verma, George D. Verros, Raj Kumar Arya

**Affiliations:** 1 Department of Chemical Engineering, Dr. B. R. Ambedkar National Institute of Technology, Jalandhar 144011, Punjab, India; sarojiniv.ch.22@nitj.ac.in; 2 Laboratory of Polymer and Dyes Chemistry and Technology, Department of Chemistry, Aristotle University of Thessaloniki (Auth), 54124 Thessaloniki, Greece; gdverros@gmail.com

**Keywords:** polyvinyl alcohol (PVA), bentonite, drying kinetics, waterborne coatings, residual solvent, film thickness, coating formulation, Simulation

## Abstract

This study explores the drying kinetics and film formation behavior of polyvinyl alcohol (PVA)-based and PVA–bentonite composite coatings with initial thicknesses of approximately 2500 µm and 2000 µm. Four coating formulations were investigated, varying in PVA concentration and presence of bentonite as an inorganic filler. The drying process was monitored through changes in solid concentration, residual solvent content, and film thickness over time. Results revealed that coatings with higher PVA content exhibit slower drying rates, due to the transition from evaporation-controlled to diffusion-limited mechanisms, attributed to polymer densification and reduced solvent diffusivity. In contrast, coatings incorporating bentonite dried more rapidly despite their similar or higher total solids content, indicating a beneficial role of bentonite in facilitating moisture transport. Thinner coatings demonstrated faster drying but retained the characteristic mechanistic transitions observed in thicker films. A simple realistic model to simulate the drying rate was also proposed. Overall, the study highlights the significant influence of formulation variables on drying behavior and final film properties, offering valuable guidance for the design and optimization of waterborne coatings in industrial applications.

## 1. Introduction

Polymeric coatings are protective layers composed of polymers, which are long chains of molecules linked together. These coatings are widely employed across various industries, including automotive, aerospace, and construction, due to their ability to enhance the lifespan, appearance, and utility of surfaces [1]. The primary function of polymeric coatings is to provide a protective barrier against corrosion, weathering, chemical attack, and mechanical abrasion. Additionally, they offer several other advantages, such as UV resistance, waterproofing, and excellent adhesion properties [2]. Application techniques vary depending on the desired coating thickness and properties, and may include spraying, dipping, or brushing [3]. Following application, the coating undergoes a crosslinking or setting process, forming a durable and adherent film on the base material through chemical and mechanical bonding. Beyond protection, polymeric coatings can significantly improve the esthetic appeal of the coated object. The polymers used in these coatings can be derived from natural sources, such as cellulose, or synthesized materials like polyethylene or epoxy. This diversity allows for the customization of coating properties, including flexibility, hardness, and environmental friendliness, to suit specific applications [4]. Ongoing research and development on polymeric coatings focuses on developing more efficient and effective methods for protecting and beautifying various materials, with applications spanning numerous fields.

Polymeric coatings can be broadly categorized into two primary types: organic and inorganic [5,6]. Organic polymeric coatings are derived from carbon-based polymers, such as polyethylene, epoxy, and polyurethane. These coatings are widely utilized, due to their inherent flexibility and excellent resistance to corrosion. Inorganic polymeric coating systems, on the other hand, are composed of compounds that do not contain carbon, including silicates, ceramics, and certain metal oxides. These coatings offer enhanced corrosion and wear protection, making them particularly suitable for high-temperature applications. Moreover, they exhibit superior resistance to chemical degradation and UV radiation.

Fillers and binders are crucial additives in polymeric coatings, significantly influencing their properties and performance [7]. Fillers, such as clay, talc, calcium carbonate, and silica, are incorporated into the polymer matrix to enhance mechanical properties, durability, and cost-effectiveness [8,9]. For example, clay improves barrier properties, enhancing resistance to moisture, gases, and chemicals. It also enhances mechanical properties, thermal stability, and substrate adhesion [10]. Fillers also influence coating rheology, enabling easier application and smoother finishes [11]. Binders, or resins, are the film-forming components that encapsulate the filler particles and provide the coating’s structural integrity. They determine key properties such as adhesion, flexibility, and environmental resistance. Common binder types include epoxy, acrylic, polyurethane, and polyester resins, each with unique characteristics [12]. Epoxy resins, for instance, exhibit excellent adhesion, chemical, and mechanical properties, making them suitable for industrial and protective coatings. Acrylic resins offer excellent weather resistance, making them ideal for exterior applications. The optimal ratio of filler to binder is critical for achieving the desired coating properties [13]. The correct filler type and loading can significantly enhance performance without compromising the binder’s characteristics. Advances in materials science and nanotechnology have enabled the development of novel fillers and binders with enhanced properties. Nanocomposites, incorporating nano clays and other nanomaterials, offer exceptional barrier properties, mechanical strength, and durability at significantly lower loadings than conventional fillers. In conclusion, the judicious selection and incorporation of fillers and binders are paramount in developing high-performance polymeric coatings that meet the diverse demands of various industrial, commercial, and consumer applications.

Polyvinyl alcohol (PVA) is a synthetic, water-soluble, and biodegradable polymer derived from polyvinyl acetate [14]. It exhibits excellent mechanical and thermal properties, forming flexible and highly transparent films with good resistance to oils, greases, and solvents [15]. PVA possesses high tensile strength, low elasticity, and strong adhesive characteristics. These properties make it suitable for various applications, including packaging, where it acts as an effective barrier against oxygen and aroma, extending the shelf life of packaged foods [16]. Key applications of PVA include the paper, textiles, adhesive, and 3D printing industries. In the pharmaceutical sector, its biocompatibility enables its use in soluble capsules and medical devices, such as vascular stents. In agriculture, it is used as a seed coating to improve water absorption and germination. Other applications include water-soluble laundry bags, detergent pods, and release liners. A significant advantage of PVA is its ability to form hydrogels without the need for additional crosslinking agents through simple freezing and thawing cycles. A major limitation of PVA-based materials is their high hydrophilicity or water solubility [16].

Bentonite clay, primarily composed of montmorillonite, exhibits unique expansive and absorptive properties [17]. Montmorillonite can swell to several times its original volume upon contact with water, making it highly effective for sealing off water. When mixed with water, bentonite forms a tightly adherent, rigid skin, making it ideal for sealing gaps, cracks, and joints to prevent water infiltration and subsequent damage [18]. This unique characteristic stems from the flowable, microporous structure of the material, which enhances adhesion and surface properties. Furthermore, bentonite offers excellent chemical stability, resisting the effects of acids, alkalis, and organic solvents [19]. It also exhibits good thermal stability over a wide temperature range. Bentonite is an environmentally friendly and non-toxic material with a wide range of applications across various industries, including construction, agriculture, personal care, and medicine. In construction, it is widely used for soil stabilization and waterproofing foundations [18]. In the personal care industry, it is a common ingredient in facial masks [20]. In pharmaceuticals, it has an application in controlled-release formulations [21]. In the food packaging industry, bentonite acts as an effective barrier material, preventing moisture and oxygen ingress and thereby extending the shelf life of packaged foods [22].

Celeizabal et al. reported that PVA’s incorporation into composite membranes, particularly with chitosan (CS), enhances their properties, such as reducing brittleness and increasing toughness. PVA’s water solubility also facilitates the formation of fabric composites, improving the mechanical strength of coatings on metallic implants [23]. Mishra et al. reported that XRD analysis of the CS/PVA composite coating revealed good polymer compatibility. This was evidenced by the intercalation of PVA chains within the CS matrix, leading to a suppression of the crystalline phase of CS and the formation of strong intermolecular interactions [24]. Jordan et al. studied that PVA-based coatings had applications in the selective coating of various forms, including pharmaceutical tablets, nutritional supplements, food confectionery, and agricultural seeds [25]. Kuca et al. demonstrated the efficiency of PVA-based coatings to enhance barrier properties such as water, grease, and oil resistance [26]. Shen et al. studied that PVA excels as a paper coating due to its strong film-forming and barrier properties, effectively sealing gaps and minimizing molecular permeation [27]. Oh et al. reported that PVA coatings increase particle toughness and strain, leading to higher CCT compared to CMC. The dense, tough PVA layer absorbs drying stress energy, preventing crack formation [28]. Zhang et al. studied that Heat treatment reduces PVA’s hydrophilicity by decreasing the number of hydroxyl groups, improving its corrosion resistance [29]. Dhaheer et al. reported that PVA coatings enhances clay brick durability by reducing porosity and water permeability, improving strength and extending service life [30]. Sharma et al. demonstrated that surfactants, like fluorosurfactants, in PVA-water coatings reduce solvent, improve drying, and prevent phase separation, leading to denser coatings, lower drying losses, and improved energy efficiency [31]. Maged et al. studied that bentonite, a smectite clay, is primarily composed of montmorillonite with a layered T-O-T structure. Isomorphous substitutions within the layers create a net negative charge. The interlayer space, containing exchangeable cations, allows for significant swelling and interaction, enabling the formation of a strong barrier [32]. Liang et al. reported that bentonite’s high surface area and cation exchange capacity make it suitable for coating applications, particularly in papermaking, where it significantly influences coating rheology [33]. Azha et al. studied that a novel bentonite-modified composite adsorbent coating exhibited high dye removal efficiency and can be easily separated [34]. Akhtar et al. studied that the synergistic combination of bentonite and polymers in coatings enhances overall performance by leveraging the unique properties of both components [35]. Hager et al. revealed that bentonite clay significantly enhances nuclear waste shielding by minimizing radionuclide migration through its low permeability [36]. Sorocina et al. reported that bentonite particles form complexes with pigments, enhancing coating stability and increasing viscosity, thereby improving overall coating efficiency [37]. Hebbar et al. reported that bentonite addition enhances membrane hydrophilicity, porosity, and water uptake, leading to increased permeation and fouling. Rejection mechanisms for heavy metals and humic acids vary with bentonite content [38]. Shaarawy et al. studied that bentonite, with its acid-resistant nature and unique aluminosilicate structure, was selected as the most suitable material for adsorbing onto the graphene oxide layer [39]. Kaur et al. depicted that bentonite clay significantly enhances the adhesive strength at the interface between the expandable graphite-based intumescent coating and the substrate steel [40].

Drying is essentially a simultaneous heat and mass transfer process in which moisture is removed from a solid by evaporating into a surrounding gas phase [41]. For solution-cast or dispersion-based coatings, the critical step of solvent removal—commonly referred to as drying—is paramount. Although often underestimated as a simple evaporation process, drying profoundly influences the final structure and properties of the polymeric film.

Drying of polymeric coatings is a complex phenomenon, involving the transformation from a dilute solution to a viscous state and ultimately into a dried solid film. In the initial stages, drying is externally controlled by evaporation of solvent from the coating surface. As solvent concentration decreases, the process transitions into a diffusion-controlled regime, which is considerably slower due to the reduced free volume available for solvent diffusion within the polymer matrix. Under quiescent drying conditions, the process remains mild, continues for longer periods, and generally allows greater solvent removal. In contrast, under high air flow, a mismatch between convective and diffusive mass transfer occurs, causing the drying rate to plummet by several orders of magnitude [42]. Practically, drying stops when a glassy layer forms at the surface, entrapping residual solvent beneath and near the substrate. This can lead to blister formation, residual solvent-induced defects, and, in some cases, phase separation.

Zhang et al. [43] investigated the drying kinetics of polymer solutions and identified three distinct stages: (i) an initial constant-rate period dominated by free solvent evaporation, (ii) a falling-rate period characterized by reduced evaporation due to increasing diffusion resistance, and (iii) a final stage where solvent removal becomes strongly diffusion-limited. The transition between these stages is strongly dependent on polymer concentration, solvent properties, and environmental conditions.

Several studies have also focused specifically on the drying behavior of PVA solutions. Fortu et al. [44] examined the influence of molecular weight and degree of hydrolysis on drying kinetics, concluding that higher molecular weight slowed down drying due to increased viscosity and reduced solvent mobility. Similarly, Chousidis et al. [45] demonstrated that increasing PVA concentration prolonged drying times and significantly affected the crystallinity and mechanical properties of the resulting films.

The incorporation of clay minerals like bentonite into polymer matrices has been explored as a strategy to enhance various properties. Wypych et al. [46] and David et al. [47] reviewed polymer–clay nanocomposites, highlighting the significant improvements in mechanical strength, barrier properties, and thermal stability achieved through proper clay dispersion. Sapalidis and Kanellopoulos [48] specifically studied PVA–clay nanocomposites and reported enhanced crystallinity, reduced water permeability, and improved mechanical properties compared to neat PVA films.

Researchers have extensively investigated the characteristics and potential applications of clay-based polymeric coatings. Sapalidis examined the characteristics of PVA–bentonite clay nanocomposite films correlated to polymer-clay interactions [49]. Aliar et al.’s study investigates an environmentally friendly semi-interpenetrating polymer network (semi-IPN) nanocomposite of PVA–alginate/bentonite synthesized for methylene blue adsorption from aqueous solutions [50]. Chandio et al. have studied that a novel nano-clay/PVOH coating was developed for packaging, exhibiting low moisture permeability (2.8 × 10^−2^ g·cm/m^2^·day), high transparency (>90%), and excellent flexibility withstanding 10,000 bending cycles [51]. Yu et al. have studied that PVA-PCN (polymer-clay nanocomposite) coatings showed increased storage moduli with higher clay loading, but optical clarity decreased, especially at high loadings, due to clay scattering. PVA molecular weight also decreased with increasing clay content [52].

Mathematical modeling of the drying process has been approached through various theoretical frameworks. The Fick’s law of diffusion has been commonly applied to describe the drying kinetics of polymer films, as demonstrated by Vinjamur and Cairncross [53]. In 2004, Wong et al. [54], provided valuable information for optimizing industrial processes that rely on drying polymer films, such as those in the pharmaceutical and food industries. The model can predict critical film properties over time, such as thickness, solvent removal rate, and crystallinity. Alternatively, empirical models such as the Page model, Henderson–Pabis model, and Lewis model have been used to fit experimental drying data, allowing for the determination of kinetic parameters [55]. Regarding the drying behavior of polymer–clay composite coatings, fewer comprehensive studies exist. Sirousazar et al. [56] investigated the drying kinetics of PVA–montmorillonite composite films and observed that clay addition altered the drying rate and influenced the final film morphology. However, the systematic examination of how varying PVA concentrations and bentonite incorporation affect the drying process remains underexplored. Therefore, a deep understanding of drying mechanisms and their precise control is not merely an engineering convenience but a scientific imperative.

Simulation studies on the drying of PVA films can be broadly categorized into six modeling approaches: diffusion-based mathematical models incorporating crystallization [54], surface segregation and gel-layer formation models [57], lumped-parameter models such as the Reaction Engineering Approach (REA) [58], empirical models including the Hill equation [59], experimental diffusivity determination approaches [60], and wrinkling and instability models [61].

Among these, diffusion-based models have been particularly effective in capturing the drying behavior of semicrystalline PVA films. Wong et al. [54] developed a rigorous framework by integrating solvent diffusion, crystallization kinetics, and film shrinkage using the Vrentas–Duda model for multicomponent diffusion. Their study highlighted how crystallinity evolves during drying, a factor that critically determines the final structure and mechanical performance of the films.

Complementary to this, surface segregation phenomena have been modeled by Zhang et al. [57], who presented a 1D description of drying in PVA–water–alumina slurries. Their work incorporated diffusion, capillary migration, and back-diffusion, revealing how PVA tends to accumulate at the surface to form a gel layer during the constant-rate drying stage. This model not only aligned well with experimental observations but also explained the early inhomogeneities frequently observed in ceramic coatings.

In contrast to these diffusion-driven models, Putranto et al. [58] employed a lumped-parameter strategy using the Reaction Engineering Approach (REA). Applied to thin PVA/glycerol/water films under convective and infrared (IR) drying, the REA simplified complex kinetics into parameters such as apparent activation energy. Their introduction of an “equilibrium activation energy” significantly improved predictions for IR-assisted drying, achieving strong agreement with the experimental data.

For more practical applications, especially in the pharmaceutical sector, empirical approaches have also been explored. A Hill-type model was successfully applied to hydroxypropyl methylcellulose (HPMC)/PVA blend films [59], capturing the two-phase drying process—an initial constant-rate period followed by a falling-rate stage—using only three parameters. Owing to its simplicity and predictive power, this approach is well-suited for rapid screening of film formulations.

To further refine model predictions, experimental determination of drying parameters has been conducted. Asoltanei et al. [60] derived diffusion coefficients for PVA–water systems through drying rate measurements combined with inverse modeling. Such experimentally obtained diffusivity values provide essential inputs for both mechanistic and empirical models, strengthening their accuracy and reliability.

Beyond mass transport, mechanical instabilities during drying have also been examined. Gao et al. [61] proposed a geometry-driven model for thin PVA soap films, demonstrating that evaporation-induced osmotic gradients generate compressive stresses, ultimately causing dynamic wrinkling. This study established a vital link between drying-induced stresses, mechanical deformation, and mass transport phenomena in ultrathin films.

The key effects of drying from PVA–water films are summarized as follows [62,63,64]:

**A. Removal of Free Water and Plasticization Effects**

○ *During drying, water acts first as a plasticizer:* it reduces the glass transition temperature (Tg) of PVA, increases chain mobility, and allows conformational rearrangements. As water is removed, chain mobility decreases, promoting crystallization and stronger intermolecular (or interchain) hydrogen bonding.
○ This change from plasticized to more rigid matrix increases stiffness and strength but can reduce elongation at break or toughness, especially if drying is very thorough.

**B. Morphology and Microstructure Evolution**

○ *Crystallization, orientation, and phase separation:* As water content falls, PVA chains crystallize more; residual water can disrupt or reduce crystallinity. In fiber or filler-reinforced systems, drying can pull PVA chains toward reinforcement surfaces, affecting interfacial zones.
○ *Formation of voids, micro-voids or porosity:* Shrinkage accompanying water removal can lead to microcracking or voids if the drying is not uniform or if internal gradients of water concentration exist. These voids act as stress concentrators.
○ *Residual stresses:* Differential shrinkage (e.g., from surface to core, or near fibers/fillers versus bulk) can lock in internal stresses, which may lead to warping, cracking, or failure under load or over time.

**C. Interfacial Bonding**

○ As water is removed, hydrogen bonding or other interactions between the PVA matrix and reinforcements (e.g., fibers and nanoparticles like graphene oxide) strengthen. In some studies, dehydration improved adhesive bonding and load transfer.
○ However, if water is trapped (e.g., in pores) or drying is too rapid (causing skin layers), the interface may be weakened or have weak boundary layers.

**D. Mechanical Properties: Strength, Stiffness, and Toughness Trade-Offs**

○ Strength and modulus tend to increase with more thorough drying (lower residual water), thanks to better packing, higher crystallinity, stronger interchain and matrix–reinforcement bonds.
○ Ductility/toughness often decreases; fracture strain or elongation decreases with drying, especially beyond a certain low residual water threshold.
○ There may be optimal water content/residual moisture that balances toughness and strength.

**E. Thermal and Dimensional Stability, Durability**

○ With less residual water, thermal stability improves (less risk of hydrolytic or moisture-induced degradation).
○ Dimensional stability under changes in humidity is better when drying is well controlled.
○ Long term durability may be enhanced, but over-drying or very fast drying can introduce micro defects that reduce fatigue or impact resistance.

Building upon these diverse insights, the present work explores the drying kinetics of PVA–bentonite coatings. The incorporation of bentonite into PVA offers a synergistic enhancement of drying behavior, mechanical strength, and environmental resistance, while also reducing cost and improving sustainability. However, to the best of our knowledge, residual solvent analysis in PVA–bentonite coatings has not yet been reported. Consequently, a major objective of this study is to develop a simple yet realistic approach to calculate drying rates, which are crucial for effective dryer design and process control.

## 2. Materials and Methods

Polyvinyl alcohol was provided by Lobachemie, Mumbai, Maharashtra, India (Density: 1.19 gcm^−3^, Molecular weight: 86.09 g/mol), bentonite clay was provided by Akemi minerals, Ahmedabad, Gujarat, India (Density: 2.2–2.8 gcm^−3^, Molecular weight: 180.06 g/mol) and distilled water (Density:1 g/cm^−3^, Molecular weight:18.02 g/mol). All chemicals were utilized as provided without any further purification.

Solution casting is a fundamental technique for preparing clay-based polymeric films. This method involves dissolving polymeric in a volatile solvent or solvent mixture to achieve a uniform and low-viscosity solution. The solution is then applied to a substrate, either by spreading or pouring into a mold. Depending on the application, the process can be batch or continuous, with the substrate fixed or mobile. Solution casting is widely used in producing films for various applications, including electronics, medicine, and optics. This technique enables the formation of uniform films with excellent optical clarity.

The preparation of clay-based polymeric coatings involves meticulous steps to ensure accurate measurements, proper mixing, and thorough data recording. Initially, precise amounts of PVA and bentonite are weighed separately according to their desired concentrations. Distilled water is used to achieve the target solution weight. Subsequently, PVA is dissolved in distilled water, followed by the gradual addition of bentonite under continuous stirring to ensure uniform dispersion. Sonication is employed to enhance homogeneity, and the mixture is stirred for an additional 30 min to ensure complete phase mixing. The mixture is then allowed to stand for a week to facilitate thorough interaction between PVA and bentonite. Subsequently, the solution is pipetted onto metallic circular sample holders of varying volumes to create films. Careful attention is given to minimize air bubbles and ensure uniform film distribution during this transfer process. Solvent evaporation from the coating after application resulted in a gradual weight loss. The sample weight was continuously monitored using a Radwag AS 82/220 X2 Plus analytical balance (Radom, Poland) with an accuracy of ±0.0001 g. Data acquisition continued until no significant weight change was observed between consecutive measurements, indicating the completion of the drying process. All coatings were dried under natural convection conditions at 25 ± 1 °C, unless otherwise specified. Four concentrations were considered:

Type 1—PVA: 5.05 wt% and water: 94.95 wt%
Type 2—PVA: 9.97 wt% and water: 90.03 wt%
Type 3—PVA: 14.95 wt% and water: 85.05 wt%
Type 4—PVA: 5.08 wt%, Bentonite: 5.03 wt%, and water: 89.89 wt%

The coating thickness at any time was calculated using volumetric calculation. The volume of entire solution was divided by the cross sectional area of the coating to obtain the instantaneous coating thickness. The changes in the lateral side of the coating were assumed negligible because of no mass transfer from any other side except top. Coating calculation procedure is explained in the Section Coating Calculations.

The dried films were subjected to SEM-EDS analysis to study the morphology and elemental analysis of dried films.

### Coating Calculations

Let the mass of solution in any time be “*m*” of polymer solution Type 1, having 5.05% polymer and 94.95% solvent, respectively [65].

$$\mathrm{Mass}\;\mathrm{of}\;\mathrm{polymer},\;M_{p}=m\times \frac{5.05}{100}$$

$$\mathrm{Initial}\;\mathrm{mass}\;\mathrm{of}\;\mathrm{solvent},\;M_{S}=m\times \frac{94.95}{100}$$

$$\mathrm{Volume}\;\mathrm{of}\;\mathrm{coating},\;V_{\mathrm{coating}}=\frac{M_{p}}{\mathrm{density}\;\mathrm{of}\;\mathrm{polymer}}+\frac{M_{S}}{\mathrm{density}\;\mathrm{of}\;\mathrm{solvent}}$$

$$\%\mathrm{Residual}\;\mathrm{Solvent}=\frac{\mathrm{Instantaneous}\;\mathrm{mass}\;\mathrm{of}\;\mathrm{coating}-m}{M_{S}}\times 100$$

$$\mathrm{Thickness}\;\mathrm{of}\;\mathrm{coating},\;L=\frac{V_{\mathrm{coating}}}{\mathrm{cross}\;\mathrm{sectional}\;\mathrm{area}\;\mathrm{of}\;\mathrm{sample}\;\mathrm{holder}}$$

$$\mathrm{Concentration}\;\mathrm{of}\;\mathrm{polymer}=\frac{m\times 5.05}{V_{\mathrm{coating}}}$$

$$\mathrm{Concentration}\;\mathrm{of}\;\mathrm{solvent}\;=\frac{\mathrm{Instantaneous}\;\mathrm{coating}\;\mathrm{mass}-M_{P}}{V_{\mathrm{coating}}}$$

A major task of this work is to simulate the drying rate data as above calculated. Please, note that if one quantity of the above is successfully simulated then the rest of the quantities are directly calculated. For example, by using gravimetric measurements of the drying rate, the thickness of the solution at any time is calculated by applying the equations previously described in this work. On the other hand, one could directly calculate the gravimetric drying rate and the other properties by using the previously described equations from simulated thickness in reverse order. In our recent review, it was shown that for high boiling point solvents such as water, an initial thickness above 1500 μm isothermal conditions during drying is a reasonable assumption [66].

Following most workers in the field for high boiling solvents such as water, isothermal conditions were considered. The starting point to simulate the drying rate is the moving boundary equation [67], describing the thickness of solution *L* in terms of non-volatile constituents (PVA + bentonite), volume fraction ($u_{2}$), and the mass transfer coefficient *K_G_*:

(1) $$u_{2}\frac{dL}{dt}=-C_{1}\left(x_{s}-x_{\infty }\right);C_{1}=K_{G}v_{1};t=0,L=L_{0}$$

where the water mole fraction at the gas–liquid interface ($x_{s}$) is calculated in terms of partial vapor pressure of water $P_{1}$ above the solution as the following:

(2) $$x_{s}=\frac{P_{1}}{P}$$

$P_{1}$ is calculated as the following:

(3) $$P_{1}=P_{1}^{0}\exp\left(\frac{\Delta \mu_{1}}{RT}\right)$$

All the symbols are explained in the nomenclature. The volume fraction $u_{2}$ at the top of the casting solution was approximated as the following:

(4) $$u_{1}=u_{10}\exp(-At),u_{2}=1-u_{1}$$

where A is a constant.

The above equation has a clear physical meaning: The volume fraction of solvent $u_{1}$ is equal to the initial volume fraction of solvent $u_{10}$ at time t equal to zero and at large, time is equal to zero as water has left the casting solution.

The chemical potential $\frac{\Delta \mu_{1}}{RT}$ was calculated by using Flory–Huggins thermodynamics by assuming a binary solution (PVA + bentonite and water). The Flory–Huggins interaction parameter for PVA/bentonite/water was set equal to 0.5.

The Flory–Huggins parameter (χ) for PVA-water is not a single fixed value, but depends on factors like the degree of hydrolysis of the PVA and the temperature [68,69]. For fully hydrolyzed PVA, the interaction is limited, requiring high temperatures for dissolution, while the parameter becomes higher for crosslinked gels, indicating weaker polymer–water interactions. Generally, a higher χ value (often >0.5 for crosslinked gels) indicates poorer miscibility [70], while a lower value suggests good solubility and strong affinity. A precise single Flory–Huggins parameter (χ) for the ternary system PVA-water-bentonite is not readily available, as the parameter depends on the specific interactions, temperature, composition, and whether it is a binary or ternary system [68], with bentonite acting as a solid filler and water acting as a solvent for PVA. For good solvent, the Flory–Huggins interaction parameter, χ<0.5. For Theta condition, χ≈0.5, where polymer—solvent interaction just balances, polymer–polymer and solvent—solvent interactions. χ>0.5, for poor solvent, where polymer tends to precipitates or aggregates. In the present, system, PVA is completely soluble in the water but bentonite precipitates. Therefore, we have considered χ≈0.5 for this system.

The overall pressure *P* was 1 atm and the pure water vapor pressure $\left(P_{1}^{0}\right)$ was set equal to 0.0313 atm (at 25 °C). Finally, the solvent mole fraction at the bulk of air phase $\left(x_{\infty }\right)$ was set equal to zero.

This leaves two adjustable parameters: The mass transfer coefficient, $K_{G}$ and the parameter, A, to be estimated by fitting solution thickness (*L*) versus time data as previously calculated in this section.

## 3. Results and Discussion

This study investigated the influence of bentonite clay content on the drying behavior of polyvinyl alcohol (PVA) coatings. PVA–water coatings with varying concentrations (5–15 wt%) were prepared for this study. Bentonite clay was employed as a filler to reduce the use of costly PVA. The effect of filler content on the drying kinetics of the PVA–water coatings was examined and reported in this section.

**Figure 1:**
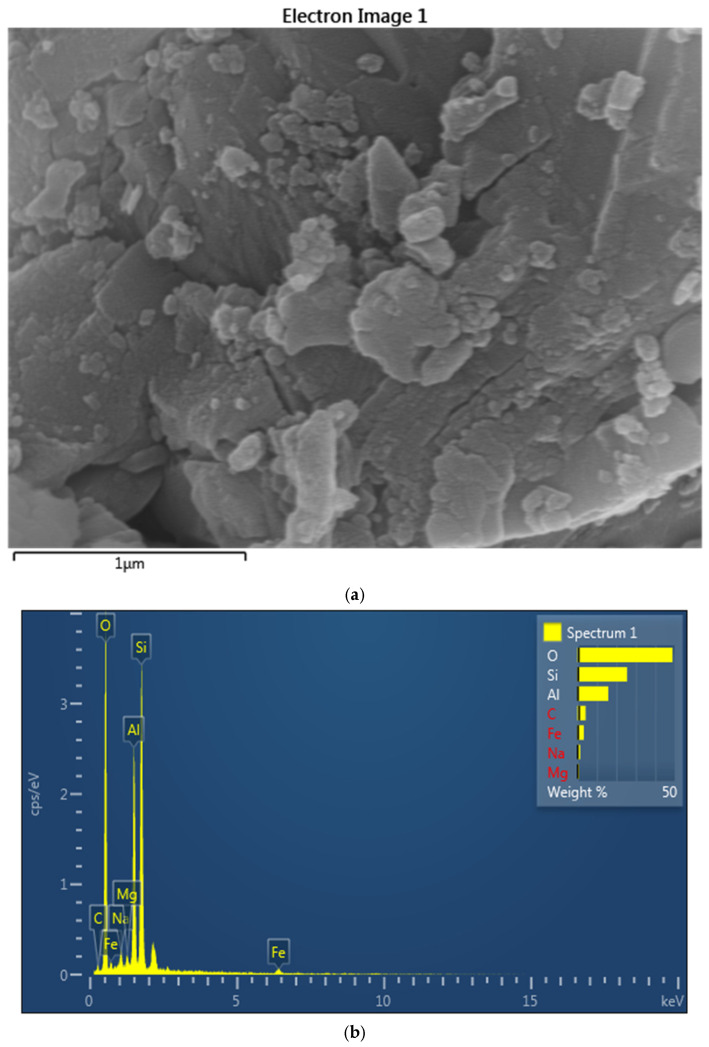
EDAX image analysis of bentonite clay: (**a**) SEM image, (**b**) EDAX maping.

Figure 1 shows the SEM image of the bentonite clay with the elemental data using EDAX.

Figure 1a depicts the SEM image of bentonite, which reveals the morphology of bentonite clay and can be categorized by its flaky and layered structure. This plate-like structure indicates the high surface area of minerals and its unique bulging properties, and is essential for the applications like adsorption, coating, and drilling muds. This image highlights the texture of the image as being highly effective in water and ions absorption. The plate shape is fundamental to bentonite’s function as a rheology modifier. When dispersed in a liquid coating, these platelets can align under shear, leading to pseudoplastic flow (thinning under stress) and subsequently form a house-of-cards structure at rest, imparting thixotropy (recovering viscosity over time). This prevents pigment settling and improves sag resistance.

Figure 1a represents the SEM graph of bentonite, which reveals its flaky, layered structure which is essential for its high surface area and unique swelling properties which enables its effective absorption of water and ions. Research was conducted by Yang et al. [71], in which they demonstrated that the activation of Na enhances the adsorption capacity of bentonite, as was evidenced by the increasing surface area and improved ion exchange properties, which make it suitable for applications in the environmental remediation and industrial processes. Similar investigation has been conducted by others [72,73], in which they investigated the effectiveness of activated bentonite for removing phenolic compounds for wastewater of olive mill.

Figure 1b shows the energy dispersive X-ray spectroscopy (EDS) spectrum of bentonite, which shows the elemental composition of the sample. The peak corresponds to oxygen (O), silicon (Si), aluminum (Al) and the smaller amounts of sodium (Na), magnesium (Mg) and iron (Fe) and carbon (C). The prominent presence of O, Si and Al indicates the primary composition of montmorillonite. The trace elements like Mg, Fe, and Na reflect the cation exchange properties of bentonite, which are crucial for its industrial application in paint and adsorption. The elemental composition confirmed by EDX is vital for quality control. High Si/Al ratios might suggest the presence of quartz impurities, which are harder and less reactive, potentially affecting film abrasion resistance or clarity. The relative abundance of Na, Ca, and K dictates the type of bentonite (e.g., sodium bentonite generally swells more than calcium bentonite), directly influencing its dispersibility and rheological efficiency in aqueous coating systems. Identifying impurities helps in predicting potential long-term stability issues, color changes, or compatibility problems within the coating formulation.

Table 1 highlights the elemental composition of bentonite; it can be seen that the most abundant elements in the matrix are oxygen, which is at 48.84 wt% (60.38% of atoms), and silicon at 25.50 wt% (17.96 atoms %). It contains 15.89 wt% of Al and 11.65 atomic %, which also provides evidence about aluminosilicate minerals and a specific type of them: montmorillonite. Carbon at 4.22 (6.95) wt%, sodium at 1.41 (1.21) wt%, magnesium at 0.83 (0.68) wt%, and iron at 3.30 (1.17) wt% are present in minor value from other phases or imperfections. This composition corresponds to natural bentonite, with uses such as adsorption and geotechnical barriers.

### 3.1. Drying of PVA—Water/Bentonite Clay of Around 2500 Microns

**Figure 2:**
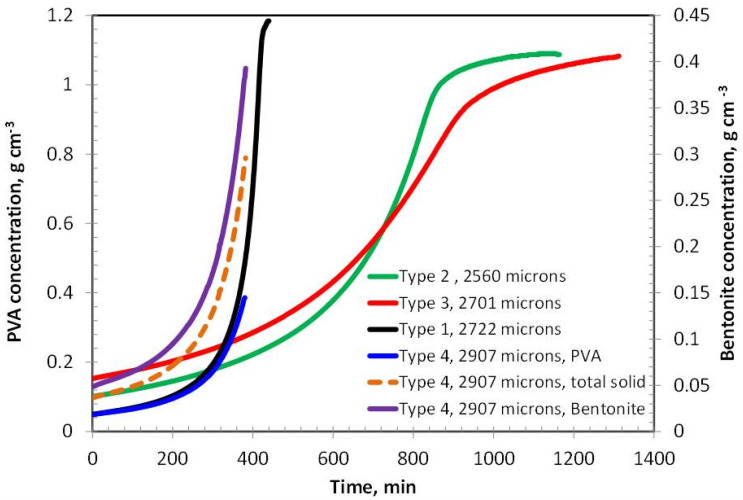
PVA/Solid concentration with time for coatings having an initial thickness around 2500 microns.

The concentration of polyvinyl alcohol (PVA) in Coating Types 2 and 3 exhibits a similar increasing trend during the initial and intermediate stages of drying, eventually reaching a plateau in the later stages, as shown in Figure 2. However, the drying rate of Type 3 is noticeably slower than that of Type 2, which can be attributed to its higher PVA content—approximately double that of Type 2. In contrast, Type 1 coating, which contains only about 5% PVA, demonstrates the fastest drying behavior among all the samples. This rapid drying may result from a predominantly evaporative mechanism with minimal diffusion resistance, as the polymer concentration is relatively low. Interestingly, Type 4 coating, which incorporates 5% bentonite and the same total solid content as Type 3 (i.e., 10% solids), dries significantly faster than Type 3. This suggests that the nature of the solid component—bentonite versus PVA—plays a crucial role in influencing the overall drying kinetics.

**Figure 3:**
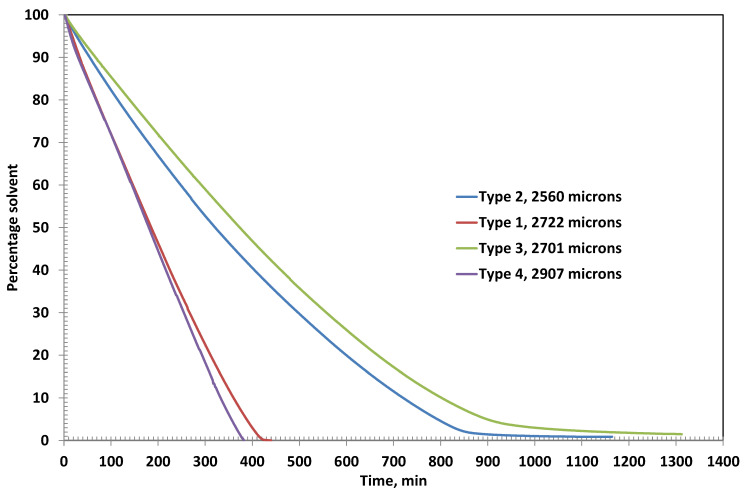
Residual solvent with time in the coatings having initial thicknesses around 2500 microns.

Figure 3 shows the residual solvent as function of time in various coatings. Type 2 and Type 3 coatings show evaporative and diffusion-controlled drying, as evident from the slope changes in the residual solvent curves at 800 min and 900 min, respectively. The amount of corresponding residual solvent is 4.59% and 4.93%, respectively. The ultimate residual solvents left are 0.88% and 1.46%, respectively. The results are in theoretical agreement because a higher polymer percentage may retain a higher percentage due to reduced free volume.

The drying behavior of Coating Types 1 and 4 is governed entirely by external mass transfer mechanisms, as indicated by the linear profiles of their residual solvent curves. This linearity suggests that the drying process in these coatings is primarily evaporation-controlled, with negligible influence from internal diffusion resistance. At the end of the drying period, the residual solvent content in Type 1 and Type 4 coatings is remarkably low—0.02% at 442 min and 0.07% at 384 min, respectively—highlighting their efficient solvent removal.

In contrast, Coating Types 2 and 3 exhibit a transition from evaporation-controlled to diffusion-controlled drying, as evidenced by the distinct change in the slope observed in their residual solvent curves at approximately 800 min and 900 min, respectively. At these inflection points, the residual solvent contents are 4.59% for Type 2 and 4.93% for Type 3. The ultimate residual solvent levels decrease to 0.88% and 1.46%, respectively, by the end of the drying process. These results are consistent with theoretical expectations, as higher polymer concentrations in Types 2 and 3 likely reduce the free volume within the film matrix, thereby impeding solvent diffusion and prolonging the drying duration.

**Figure 4:**
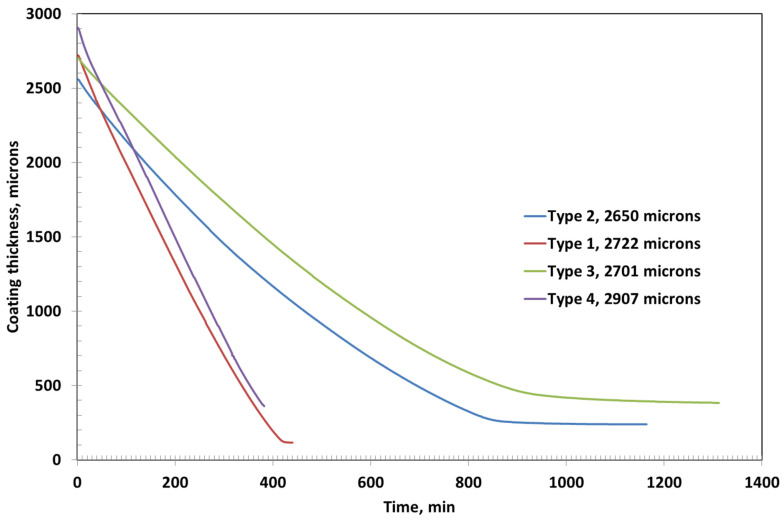
Coating thickness as a function of time for various coatings with initial coating thicknesses of around 2500 microns.

The initial coating thicknesses for Type 1, Type 2, Type 3, and Type 4 were measured to be 2722 µm, 2560 µm, 2701 µm, and 2907 µm, respectively. Among these, Type 3 coating required the longest drying time of approximately 1314 min, followed by Type 2, which dried in around 1164 min, as shown in Figure 4. In contrast, Type 1 and Type 4 coatings exhibited significantly shorter drying durations of about 442 min and 384 min, respectively—less than one-third of the time required by Type 3.

The final (ultimate) thicknesses of the dried coatings were substantially reduced due to solvent evaporation. Type 1 coating reached a final thickness of 116 µm, while Type 2 and Type 3 coatings resulted in final thicknesses of 239 µm and 384 µm, respectively. Type 4 coating, despite its high initial thickness, dried down to 361 µm. These variations in drying time and final thickness can be attributed to differences in formulation—particularly polymer concentration and the presence of fillers like bentonite—which influence solvent retention, diffusion rates, and film compaction during the drying process.

### 3.2. Drying of PVA—Water/Bentoite Coatings of 2000 Microns Thickness

**Figure 5:**
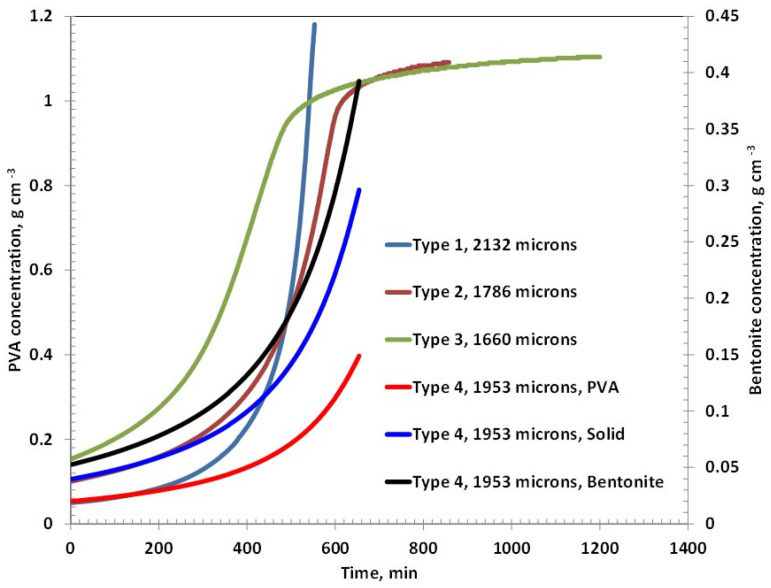
PVA/solid concentration as a function of time for various coatings of initial thicknesses of around 2000 microns.

The observed drying trends in Figure 5 closely resemble those of previous coatings with initial thicknesses around 3000 µm, though changes occur more rapidly in the present case. Among all formulations, Type 3 coatings containing approximately 15% PVA exhibit a quicker approach to the plateau phase of drying, indicating early transition to the diffusion-controlled regime. This accelerated transition is likely due to the formation of a dense, glassy polymer layer on the surface as the solvent evaporates. Once this surface layer forms, mass transfer of the remaining solvent from the interior is significantly hindered, often by several orders of magnitude, due to reduced diffusivity through the compacted polymer matrix.

In the case of the Type 4 coating, the drying profiles of PVA and bentonite appear to overlap, suggesting a uniform distribution and simultaneous evolution of both components during drying. However, despite a similar solid content to Type 3, Type 4 demonstrates the slowest overall drying behavior. This can be attributed to the high moisture retention capacity of bentonite clay, which retains water within its layered structure and prolongs the drying process. The hydrophilic nature and swelling characteristics of bentonite likely impede solvent release, resulting in extended drying times compared to purely polymer-based systems.

**Figure 6:**
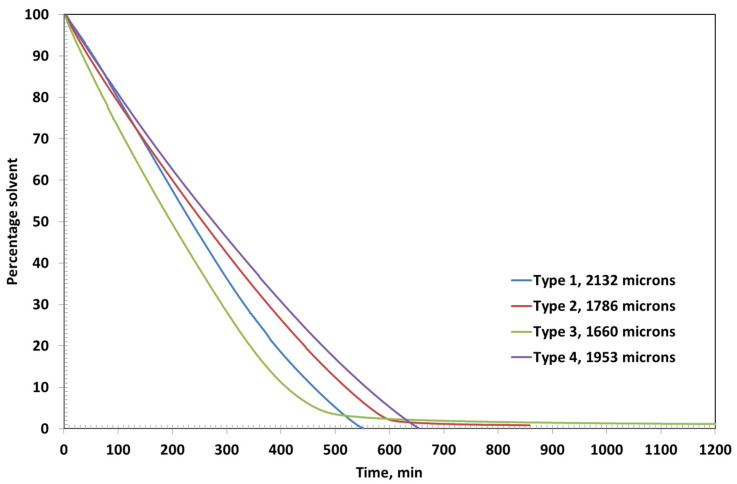
Percentage solvent as a function of time for coatings of initial thicknesses of around 2000 microns.

Type 2 and Type 3 coatings exhibit diffusion-controlled drying behavior, which is particularly evident in the later stages of the drying process, as shown in Figure 6. This is characterized by a noticeable change in the slope of the residual solvent curves, indicating a shift from surface evaporation to a slower, diffusion-limited mechanism. As the solvent content decreases and the coating film densifies, the internal mass transfer becomes the rate-limiting step, due to reduced free volume and increased polymer chain entanglement.

The final residual solvent contents further support this observation. Type 1 coating, with the lowest polymer concentration, retains only 0.03% solvent, reflecting its predominantly evaporation-controlled drying. Type 4 coating also shows efficient solvent removal, with a residual solvent content of just 0.07%, likely due to the presence of bentonite, which facilitates early water release despite its moisture-holding capacity. In contrast, Type 2 and Type 3 coatings retain higher amounts of residual solvent—0.84% and 1.15%, respectively—highlighting the significant impact of increased PVA concentration on diffusion resistance and solvent entrapment within the film matrix.

**Figure 7:**
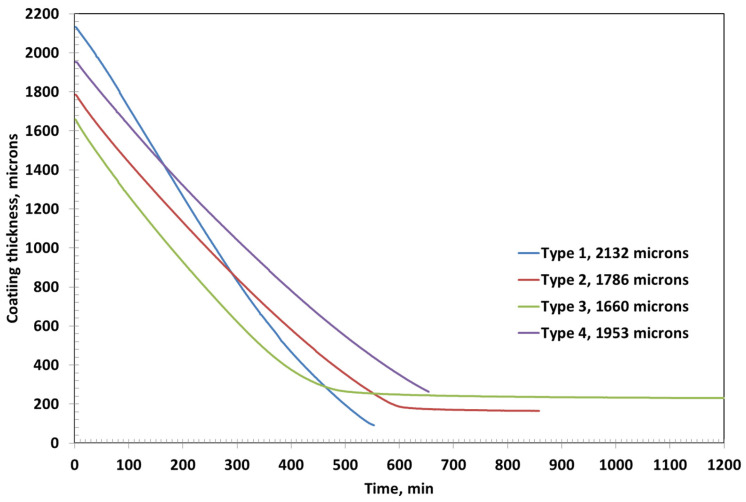
Coatings thickness as a function of time for various coatings of initial thicknesses around 2000 microns.

Among all the formulations, Type 3 coating results in the thickest dried film, which can be attributed to its highest PVA content (approximately 15%) compared to the other coatings as shown in Figure 7. The high polymer concentration likely leads to greater solid accumulation upon drying, resulting in a more substantial final film thickness. The second thickest coating is observed in Type 4, which contains bentonite clay. As a non-volatile inorganic filler, bentonite not only contributes directly to the solid content but also has a strong water retention capability due to its layered structure and hydrophilic nature. This may lead to slower water release and a comparatively thicker final film.

In contrast, Type 1 coating, with only 5% PVA and no additional fillers, yields the thinnest dried layer. The low initial solid content facilitates rapid evaporation with minimal residual buildup, resulting in a final thickness of just 91 µm. For comparison, the final dried thicknesses of Type 2, Type 3, and Type 4 coatings are approximately 165 µm, 231 µm, and 263 µm, respectively. These results clearly demonstrate the influence of the initial composition—particularly polymer concentration and filler content—on the ultimate coating thickness and drying characteristics.

The drying behavior of the coatings is strongly influenced by the formulation’s polymer and filler content. At an initial thickness of ~2500 µm (Figure 2, Figure 3 and Figure 4), Coating Type 1—with the lowest PVA content (5%)—exhibited the fastest drying rate and shortest total drying time (442 min). This is attributed to its low viscosity and high solvent mobility, enabling rapid evaporation governed primarily by external mass transfer with minimal diffusion resistance. The residual solvent profile confirms a linear drying trend, consistent with an evaporation-controlled mechanism.

In contrast, Coating Types 2 and 3 (with higher PVA contents) transitioned from evaporation-controlled to diffusion-limited drying. The inflection points at ~800 and 900 min, respectively, mark the onset of diffusion-controlled drying, likely due to densification of the polymer matrix, which restricts internal solvent transport. This transition is more pronounced in Type 3 due to its higher PVA content (~15%), resulting in the longest drying time (~1314 min). These observations are consistent with polymer physics, where increased solid content reduces free volume and solvent diffusivity.

Interestingly, despite having the same total solids as Type 3, Type 4 (containing 5% bentonite) dried significantly faster (384 min). This suggests that the presence of bentonite facilitates solvent migration—possibly due to its porous structure aiding capillary-driven flow—even though it has a high moisture-holding capacity.

When the initial coating thickness is reduced to ~2000 µm (Figure 5, Figure 6 and Figure 7), the drying behavior becomes more rapid across all formulations. The thinner films allow for quicker moisture transport and earlier transition to diffusion-controlled regimes. However, the trend in drying sequence remains consistent: Type 1 dries the fastest, followed by Types 4, 2, and 3. Type 3 again shows the most prolonged drying due to early glassy skin formation at the surface, impeding internal solvent transport.

The residual solvent content at the end of the drying period corroborates the above interpretation. For the thicker films (Figure 4), Types 1 and 4 showed minimal solvent retention (0.02% and 0.07%, respectively), confirming efficient evaporation-dominated drying. In contrast, Types 2 and 3 retained significantly more solvent (0.88% and 1.46%), highlighting the increasing impact of polymer-induced diffusion resistance.

This trend holds true even at a lower thickness (~2000 µm; Figure 7), where Type 3 retained the most solvent (1.15%) and Type 1 the least (0.03%). Type 4’s residual solvent content (0.07%) remains low, underscoring bentonite’s dual role in both moisture retention and early release via external diffusion pathways.

Final film thicknesses (Figure 6 and Figure 7) reveal the influence of solids loading and composition on film compaction. Type 3 consistently produces the thickest dried films due to its high PVA content. Type 4 follows closely, suggesting that bentonite contributes significantly to solid build-up post drying. The non-volatile nature of bentonite and its resistance to compaction enhance final thickness despite lower polymer content.

Type 1 yields the thinnest films, consistent with its low solids and rapid solvent loss, which minimizes residual solids. Type 2’s intermediate composition results in moderate thickness, highlighting the linear correlation between initial polymer concentration and ultimate film buildup.

### 3.3. Simulation Studies

**Figure 8:**
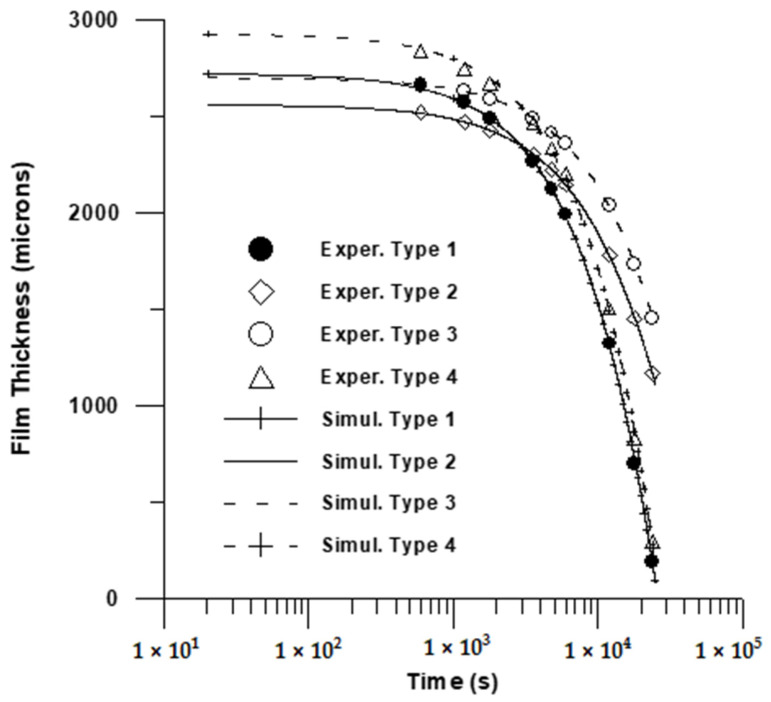
Comparison of model prediction with experimental data (2500 μm). 1 × 10^3^.

**Figure 9:**
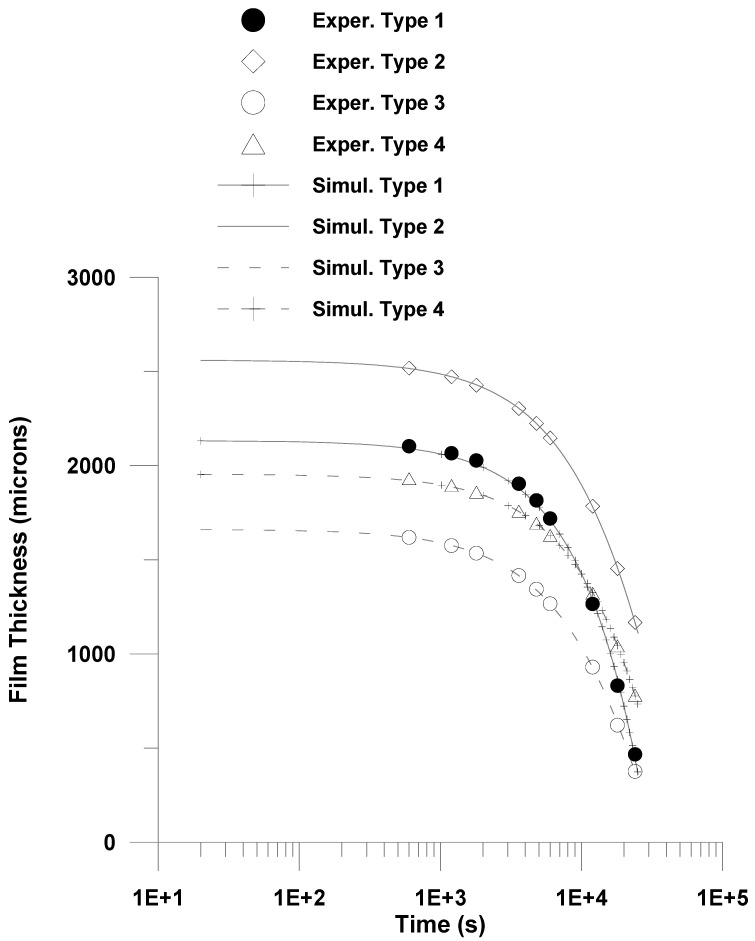
Comparison of model prediction with experimental data (2000 μm).

In this work, the moving boundary Equation (1) was numerically solved and the predictions were fitted to solution thickness data for (*L*). Two adjustable parameters were chosen: the mass transfer coefficient (Equation (1)) and the parameter *A* (Equation (4)). Standard methods of non-regression analysis [74] were utilized in this work in order to simulate experimental drying data. This comprehensive methodology permits identifying a trust region of adjustable parameters, possible indeterminate parameters, etc. The calculated values of adjustable parameters including the trust region, as well as the maximum deviation between calculated and observed values, is Illustrated in Table 2. The resulting fitting is depicted in Figure 8 and Figure 9.

A fairly good fitting is depicted in Table 1 and in both Figure 8 and Figure 9. The estimated mass transfer coefficient values were in the range 10^−4^–5.25 × 10^−4^ $kg\;m^{-2}\;s^{-1}$ almost one order of magnitude below the measured values for natural convection mass transfer coefficients for evaporation from pure water [75]. Finally, a relatively high value for solvent volume fraction at the late stage of drying was calculated in this work. This could be attributed to the fact that the calculated solvent volume fraction in this work could be viewed as an apparent volume fraction, accounting for the complex phenomena taking place in the drying of PVA film, such as crystallization, convective flows, glass transition, etc.

## 4. Conclusions

This study systematically investigated the drying behavior of PVA-based and PVA–bentonite composite coatings at two initial thickness levels (2500 µm and 2000 µm), revealing critical insights into how formulation parameters influence drying kinetics and final film properties. The findings underscore that drying is governed by a nuanced balance between polymer concentration, the nature of the solid components, and coating thickness.

Higher PVA content significantly slows the drying process due to the formation of dense polymer networks that hinder internal solvent diffusion, resulting in greater residual solvent retention and thicker final films. Conversely, coatings with bentonite—despite its intrinsic water-holding capacity—demonstrated faster drying, likely due to its porous structure and ability to facilitate moisture redistribution and surface evaporation.

Coating thickness also played a pivotal role, with thinner films drying more rapidly and exhibiting earlier transitions to diffusion-limited regimes, although the underlying drying mechanisms remained consistent across thicknesses. A simple model to simulate drying data was also developed.

These conclusions provide a foundational understanding for tailoring waterborne coating formulations where controlled drying profiles and optimized final film characteristics are essential. The insights gained can guide the design of efficient coatings in industrial applications, particularly where drying speed, solvent removal, and film uniformity are critical performance criteria.

## Figures and Tables

**Table 1:** Elemental Composition of Bentonite (Spectrum 1) by Weight and Atomic Percentage.

Spectrum 1	Line Type	wt%	Atomic %
C	K series	4.22	6.95
O	K series	48.84	60.38
Na	K series	1.41	1.21
Mg	K series	0.83	0.68
Al	K series	15.89	11.65
Si	K series	25.50	17.96
Fe	K series	3.30	1.17
Total		100.00	100.00

**Table 2:** Resulting Fitting and Estimated Parameters.

Type	L_0_ (μm)	Maximum Deviation %	A × 10^6^ (s^−1^)	−Log_10_(*K*_G_)
2500 μm				
1	2722	3.4	0.95 ± 0.15	3.75 ± 0.02
2	2560	0.8	2.38 ± 0.14	3.69 ± 0.02
3	2701	1.1	1.92 ± 0.18	3.605 ± 0.02
4	2927	4.2	2.3 ± 0.80	3.28 ± 0.02
2000 μm				
1	2132	3.2	0.29 ± 0.05	4.01 ± 0.02
2	1786	1.8	1.82 ± 0.15	3.77 ± 0.01
3	1660	1.9	4.61 ± 0.47	3.52 ± 0.02
4	1953	3.4	0.95 ± 0.16	3.75 ± 0.01

## Data Availability

The original contributions presented in this study are included in the article. Further inquiries can be directed to the corresponding author.

## Nomenclature

*A*	Adjustable parameter, s^−1^
*K*_G_	Mass transfer coefficient, kg.m^−2^s^−1^
*L*	Position of the gas–liquid interface, m
*L*_0_	Initial solution thickness, m
*M*_p_	Mass of polymer
*M*_s_	Initial mass of solvent
*P*	Total pressure at the air phase, atm
$P_{1}^{0}$	Pure solvent vapor pressure, atm
*R*	Universal gas constant, J.mol^−1^.K^−1^
*t*	Time, s
*u*_1_	Solvent volume fraction, dimensionless
*u*_10_	Initial volume fraction of the solvent, dimensionless
*u*_2_	PVA + bentonite volume fraction
*v*_1_	Partial specific volume of the solvent, m^3^.kg^−1^
*x*_s_	Solvent mole fraction at the air phase (gas-film interface)
*x*_∞_	Solvent mole fraction at the air phase (bulk)
*z*	Axial coordinate, m
